# Beyond images. ‘Titles’ between handwriting and storytelling in the works of Curzio Di Giovanni and Marco Raugei

**DOI:** 10.1017/S2045796021000469

**Published:** 2021-08-05

**Authors:** Gloria Marchini

**Affiliations:** Independent Scholar, Italy

Giving a title to a work is equivalent to giving it a name, a precise identity.

With the title we build a semantic cage that is able to influence our cognitive abilities. Words are combined with figures, sounding them out and adding meaning.

In the field of Art Brut, the use of writing is often linked to an alphabetical subversion and to a highly personal redefinition of reality: words can have a pictographic value, in other cases they contain private languages to the point of becoming exclusive ornamental motifs.

In this research the interest has shifted towards the affixing of the title, the final act in the realisation of works and the moment of total definition of a personal world, put on paper.

The two Italian authors in question, Curzio di Giovanni and Marco Raugei, are both linked to Italian workshop realities. Title, signature and date in their works are placed at the end of a compositional ‘rhythm’ and a sort of sacred ritual.

Curzio Di Giovanni (Lodi, 1957) was one of the artists of the *Atelier di Pittura Adriano e Michele* in San Colombano al Lambro, outside Milan; Marco Raugei (Florence, 1958–2003) is one of the ‘historic’ artists of *Laboratorio di Arti Espressive La Tinaia* in Florence.

Curzio Di Giovanni's unusual collection of portraits was born out of inspiration found in the pages of fashion magazines, advertising campaigns and art catalogues left to the free use of users of the Atelier in San Colombano al Lambro. As Teresa Maranzano, art historian and coordinator of the workshop's activities, explains, the idea was to offer illustrations without too many contaminations, in order to focus attention on the main forms (in this sense the fashion images worked perfectly).

Di Giovanni suffers from an autistic condition that has affected his mental and intellectual development. He entered the psychiatric rehabilitation unit of the Fatebenefratelli Centre, near Milan, at the age of 22, and from 2001 started to attend the Atelier about twice a week until it closed. He has never faithfully copied the source images; on the contrary, he has totally ignored their rules and proportional principles, bending and manipulating them to his idea of reality.

The first work he produced from a pre-existing model was a horse's head: as soon as he saw the image, he decided to draw it. Thereafter Curzio was constantly welcomed into the Atelier, something never seen before for a similar clinical case: extremely ritualistic, he found it hard to break away from his habits. Drawing, on the other hand, had managed to gain a space among the necessary activities and had been included in his ‘rhythm’: twice a week he was taken from his ward and, after a coffee, two cigarettes and the usual walk around the room, he chose a model and set to work, completing the drawing in about one to two hours.

His drawings show a total absence of hesitation: they are free of erasures. Lines, marked and decisive, define the contours of the spaces to be filled with colour. The original volumes are often filled in with flat colours and the absence of a background gives the portrait omnipotence (Peiry, 2018).

The result is a mosaic of signs, light and shadow. The idealised version of fashion icons is totally subverted, making deformation the key element of his productions.

This aspect can also be seen in the title: words are often altered, broken up, divided, elongated in a way similar to drawing. Writing is a fundamental part of Curzio Di Giovanni's production. Even before arriving at the Atelier di Pittura Adriano e Michele in 2001, Di Giovanni wrote ‘themes’ characterised by simple sentences and letters in cursive, rounded and soft (Maranzano, 2007). This heritage can be found in titles of the drawings, written in the same style.

The title, in Curzio's works, is the final aspect of the drawing ritual. Once he had had his assistants explain to him what he had just drawn, he would turn the paper over and, in a variable order, sign it, add the title and the date. The elementary, rounded calligraphy refers to a rhythm, sometimes jagged, sometimes more harmonious, which is also repeated in the handling of the individual letters: sometimes he adds a few extra ‘i's to words, sometimes he adds a macron (ō) to his name, free-running without any pattern.

As in the case of the images on the front, a process of decomposition seems to take place in the writing: certainly, ‘Un n n n Uommo con n Il Visso Rottonnddo’ (‘Man with a Round Face’) does indeed refer to the representation of a ‘Man with a Round Face’, but if the latter definition can be perfectly linked to the original image, the definition that marks Curzio is, even in the title, his personal interpretation of what has been drawn, distorted in a mannerist way in a sort of literal appropriation and alphabetical subversion. His name, dates and titles are distorted, just as the starting reality of the portrayed image.

**Fig. 1.**
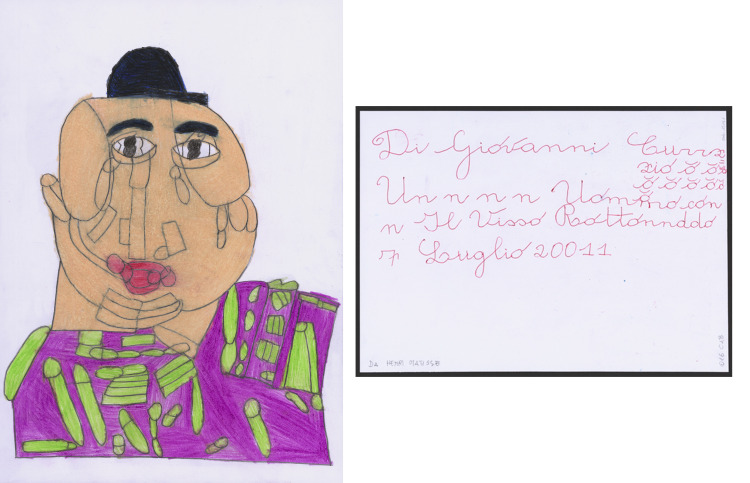
*Un n n n Uommo con n Il Visso Rottonnddo*, 2011. Photo: Sarah Baehler, Atelier de numérisation – Ville de Lausanne.

**Fig. 2.**
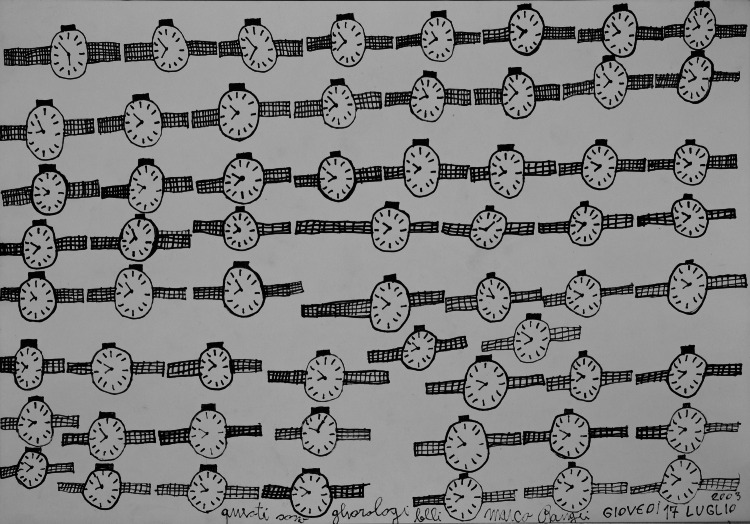
*Questi sono gliorologi belli*, 2003. Photo: Associazione La Nuova Tinaia, Firenze.

Somehow, what is visible in images is – on the back – visible (and readable) in words.

Like an unaware Apollinaire, Curzio plays with letters, fits them together, moves them like the lines of his angular portraits, distorts and enlarges them, transforming them into graphic elements, changing years (201111) and distorting months (Febbraiiiiiio) by inserting them into his personal time.

Marco Raugei also uses titles to describe the rhythmically repeated shapes in his drawings.

Raugei began to frequent *La Tinaia* in 1986, but it was not until the end of 1988 that he actually defined his language.

His works in felt-tip pen on paper are often based on the repetition of a single subject in stylised and essential forms and, occasionally, landscapes or depictions of characters in interiors. The image resulting from the reiteration – with subtle variations – in shape and size, becomes the archetype of the ‘thing’ reproduced. A primary element, represented in its essentiality.

Left-handed, he started from the lower right-hand corner of the sheet to rise in a pattern that remains compositely within margins.

Partly from memory, partly inspired by what he saw in the studio or by the suggestions of those around him, he ‘schematized’ watches, syringes, cigarettes, cans, shoes and trains with great capacity for synthesis, following a regular, rhythmic sequence. Representations are therefore in tune with titles, given by the author and placed on the back or directly in the drawing, embedded between images, sometimes hidden, sometimes more evident, as if to underline the importance of their presence.

Marco Raugei's titles highlight the author's total presence: it is as if Raugei were here with us, telling us about the work, whereas he often remained silent and shy in reality. The title seems to be dropped as an indication of an external subject, of vaguely comic-strip-like memory. It often appears just below the drawing, on the same side of the sheet.

‘Questi sono gliorologi belli’ (‘These are the beautiful clocks’), he wrote on Thursday 17 July 2003 under 65 clocks marking different times. Because those, indeed, are Raugei's clocks: repetition, an essential element of his work, allows objects to exist only visually in the concept of an almost hypnotic seriality, caused also by the rhythmic gesture of the pen which reveals, like a mantra, a path to alienation, a social disconnection and a great capacity for imagination (Thévoz, 1994).

Added to this is the inscription on the sheet, which seems to be an invitation to read the image, to name objects, to say what the image shows, to establish, in the visual field, the verbal plane. A sort of invitation to sonorise the drawing, making it real.

To underline the actual existence of what is represented, a verbal indication appears: ‘these are’. ‘These’ refers to the drawing, to ‘its’ constructed and drawn reality.

Magritte, in his famous work ‘La Trahison des images*’*, recalled that ‘*Ceci n'est pas une pipe*’, to underline the difference between reality and figuration; Marco Raugei, on the other hand, seems to invite us into his world and affirms its absolute truth.

Outside or inside the story, both authors decide to add a further, unconscious explanation of what is told through their images. The works thus acquire an additional value, thanks to the title: an irregular and varied oral narration based on a personal rhythm. Title and subject become one, for the singular truths of the two authors, who not only start from reality, but also interpret its content themselves.

## About the author

**Gloria Marchini** is a graphic designer and an independent researcher of Outsider Art. She is specialised in Digital Applications for Art. In 2014 she started the blog outsiderartnow.com, to contribute to the diffusion of Outsider Art in Italy.

Carole Tansella, Section Editor
